# Characterizing the impacts of public health control measures on domestic violence services: qualitative interviews with domestic violence coalition leaders

**DOI:** 10.1186/s12889-023-16471-4

**Published:** 2023-09-05

**Authors:** Jennifer A. Horney, Ruth Fleury-Steiner, Lauren C. Camphausen, Sarah A. Wells, Susan L. Miller

**Affiliations:** 1 Epidemiology Program, University of Delaware, 100 Discovery Blvd, Room 731, Newark, DE 19713 USA; 2 Department of Human Development and Family Sciences, University of Delaware, 118 Alison Hall, Newark, DE 19716 USA; 3 Department of Sociology and Criminal Justice, University of Delaware, 335 Smith Hall, Newark, DE 19716 USA

**Keywords:** Domestic violence, COVID-19, Intimate partner abuse, Disaster, Pandemic

## Abstract

**Background:**

Prior to the availability of pharmaceutical control measures, non-pharmaceutical control measures, including travel restrictions, physical distancing, isolation and quarantine, closure of schools and workplaces, and the use of personal protective equipment were the only tools available to public health authorities to control the spread of COVID-19. The implementation of these non-pharmaceutical control measures had unintended impacts on the ability of state and territorial domestic violence coalitions to provide services to victims.

**Methods:**

A semi-structured interview guide to assess how the COVID-19 pandemic impacted service provision and advocacy generally, and how COVID-19 control measures specifically, created barriers to services and advocacy, was developed, pilot tested, and revised based on feedback. Interviews with state and territorial domestic violence coalition executive directors were conducted between November 2021 and March 2022. Transcripts were inductively and deductively coded using both hand-coding and qualitative software.

**Results:**

Forty-five percent (25 of 56) of state and territorial domestic violence coalition executive directors representing all 8 National Network to End Domestic Violence (NNEDV) regions were interviewed. Five themes related to the use of non-pharmaceutical pandemic control measures with impacts on the provision of services and advocacy were identified.

**Conclusions:**

The use of non-pharmaceutical control measures early in the COVID-19 pandemic had negative impacts on the health and safety of some vulnerable groups, including domestic violence victims. Organizations that provide services and advocacy to victims faced many unique challenges in carrying out their missions while adhering to required public health control measures. Policy and preparedness plan changes are needed to prevent unintended consequences of control measure implementation among vulnerable groups as well as to identify lessons learned that should be applied in future disasters and emergencies.

**Supplementary Information:**

The online version contains supplementary material available at 10.1186/s12889-023-16471-4.

## Introduction

The United Nations’ UNWomen has called domestic violence a “shadow pandemic” during the COVID-19 pandemic, pointing to the simultaneous impacts of reduced capacity in health services, shelters, and helplines as the pandemic intensified [1]. In the U.S., the National Domestic Violence Hotline, a source for victim resources and safety planning services, reported increases in the use of online chat services (+ 19%), in the use by victims identifying as Asian (+ 24%), and in the need for protective / restraining order assistance (+ 40%) [2].

Research and media reports on domestic violence during the pandemic have consistently described increases in both reporting and unmet needs. Public health control measures, like stay-at-home orders, isolation and quarantine, and closure of schools, courts, and non-essential businesses often left victims isolated with abusers [3]. In a systematic review and meta-analysis of reported domestic violence incidence before and after the initial COVID-19 lock down period, both global and U.S.- based studies showed statistically significant increases in domestic violence during the initial stay-at-home order period [4]. The initial lockdown period was also a time during which abusers could increase their exercise of power and control over victims due to the implementation of control measures like social and physical distancing [5].

Public health control measures typically include pharmaceutical and non-pharmaceutical interventions designed to slow or stop the spread of a communicable disease. However, for at least the first year of the pandemic, only non-pharmaceutical interventions were widely available. During that time, the impact of these control measures on both victims and service providers evolved along with changes in the principal focus of the public health response. For example, agencies developed new solutions for victims, including virtual services, while at the same time implementing new supports to enhance advocates’ resilience [6]. The following paper adds to our understanding of the impacts that non-pharmaceutical control measures – implemented in response to the COVID-19 pandemic by public health authorities – had on the provision of domestic violence services and service providers by elaborating on new risk factors for violence and abuse as well as changes to access to supports [7–9].

## Methods

An seven-question key informant interview guide was developed based on issues raised in the published literature and initial discussions with several key informants about (1) how the COVID-19 pandemic affected survivor-centered and empowerment-focused service provision, and (2) barriers created by public health control measures that impacted advocacy and other system-level services. The semi-structured nature of the interview guide (See Appendix 1) allowed for additional probes and queries to explore underlying assumptions and open opportunities for ideas to be shared [10, 11]. To enhance trustworthiness, all the interviews were conducted by two members of the research team who are current or former state domestic violence coalition board members. Their familiarity with advocacy services and understanding of domestic violence allowed for shared language and a stronger establishment of trust and rapport between respondents and interviewers, thus facilitating respondent participation. The study was approved by the University of Delaware’s Institutional Review Board (1597257–2). Informed consent was obtained from each participant prior to the start of each interview.

### Recruitment and sample

Initial email invitations were sent to state and territorial domestic violence coalition executive directors in all 56 U.S. States and Territories to introduce the research project and schedule interviews. Executive directors were contacted up to five additional times over a 4-month period in early 2022. After some executive directors completed interviews, they assisted in recruitment by reaching out to other states’ directly and allowing the research team to use their names to gain entrée. The final sample included 25 of 56 coalitions (45%), representing all 8 of the National Network to End Domestic Violence (NNEDV) regions (New England, Mid-Atlantic, Gulf States, Southern States, Upper Midwest, Lower Midwest, Mountain States, and West Coast). Interviews were conducted over Zoom (San Jose, CA), which provided recordings and verbatim transcripts that were reviewed and edited for clarity by another member of the research team.

### Coding and analysis strategy

The semi-structured interview guide provided flexibility for participants to raise issues that were not specifically asked about. Interviews lasted an average of 44 min (Range: 27–60 min). After each interview, researchers wrote notes that highlighted issues raised and reflections about the interview and the participants’ insights [12]. Thus, both inductive and deductive themes emerged with deductive themes theoretically/empirically identified in prior research regarding coalitions’ engagement with the COVID-19 pandemic and inductive themes identified by key informants. Following a grounded theory tradition, we employed the constant comparison method [13, 14]. Two senior members of the research team coded the data; one used hand-coding and the other using Dedoose (Manhattan Beach, CA) software. Discrepancies in coding were rare and resolved through discussion. Coding continued until saturation was reached, meaning that no new themes emerged from the interview data [15, 16].

## Results

The principle non-pharmaceutical public health control measures implemented during the COVID-19 pandemic included travel restrictions, physical distancing, isolation and quarantine, closure of schools and workplaces, and the use of personal protective equipment. Impacts of the implementation of these control measures from the perspective of state and territorial domestic violence collation executive directors are summarized below.

### Travel restrictions

Many of the executive directors mentioned how affected their coalitions were early in the pandemic when domestic violence shelter staff were not classified as essential workers even though staff in similar settings, such as homeless shelters, were considered essential. As one executive director put it, “in early March 2020, our advocates were stopped on the way to work because they were violating the stay at home order because they weren't considered essential.” This was a particular problem in small states where staff may live in one state but work in another as well as in rural areas, where long travel times of “up to 9 h” were required to reach shelters.

Travel restrictions disrupted the distribution of limited and much needed supplies such as Personal Protective Equipment (PPE), cleaning supplies, diapers, and food while also limiting the access domestic violence advocates had to those utilizing their services. The inability to travel also had severe impacts on service provision because, at that time, there was no technology set up for remote work. Further, access to other services was also limited due to the inability to travel, as well as closures, with one advocate pointing out that “three of our four courthouses closed so victims and advocates had to travel 40 min to get to the only open courthouse. But they weren’t allowed to be on the road in the early days [of the pandemic].” Much of the coalition-level work also involved travel to conduct trainings, which was stopped, making it hard for organizations that depended on in-person communication and connection. As summarized by one respondent, “there was such a level of uncertainty…and it was pretty desolate the first weeks and months of the pandemic”.

### Physical distancing and restrictions on mass gatherings

All executive directors interviewed mentioned the difficulty of using congregate living situations in shelters, summing this up by saying “shelters dramatically changed [in ways that made it impossible to do] advocacy when there’s no infrastructure.” Keeping clients six feet apart meant that for “one shelter that had 30 people now has nine people…so we were turning away victims.” Shared kitchens and living spaces made it difficult to keep six feet apart. Shelters had to be reconfigured and capacity was often reduced by at least half, which was hard because shelters are nearly always full. “Our shelters had to do a lot of renovations…our shelters are already small and they had to figure out how to keep families completely separate and have space for children to do their homework because they were doing virtual school.”

The reduction in shelter space coincided with a lack of affordable housing in many areas, leading some shelters to “close down their shelter and move to their population to hotels so that (clients) could be isolated in their own spaces.” But hotel vouchers were also limited, a shortage that was more severe in rural areas with few hotels and in areas that were impacted by disasters like hurricanes or winter storms. Efforts to adhere to physical distancing guidelines also “had a rolling effect around things like case management, which was a lot harder when people (were) spread out through hotels versus in a congregate living space with staff on site, where you can interact with people.” Similar challenges were faced “with things like meal preparation and counseling support.”

Finally, adherence to physical distancing in shelters led to increased stress among both clients and staff. Worries about keeping themselves and their families safe – and adhering to COVID protocols to keep clients safe – meant that a “lot of shelter staff [left] and programs have a hard time re-hiring for shelter staff because they're the ones that are most directly exposed.” There were also financial stressors related to physical distancing. To increase opportunities for clients to spend time outside, shelters spent funds on fenced yards, playground equipment, and cameras “so that people could feel comfortable and safe outdoors.” Eventually, the fear of contracting COVID-19 “decreased the number of survivors seeking shelter” in some areas.

### Isolation and quarantine

Similar to the published literature, all executive directors expressed concerns about the impacts of isolation and quarantine on victims, pointing out that “survivors were potentially sheltering in place with a person who harms them….in unsafe environments, [with] less access to their support outside of the home, whether it was coworkers, their faith-based community, family, or neighbors.” The balance between victim empowerment and public health mandates related to isolation and quarantine was exceptionally difficult for domestic violence service providers and advocates. “All shelter programs get federal funding. And of course, the VAWA (Violence Against Women Act) and federal funding requirements say ‘everything has to be confidential, and nothing can be mandated.’ So at times we'd have shelter staff say ‘We're thinking of requiring everyone to get tested before they come in.’ And we’d say, ‘you actually can't do that because that would be mandating.’” Coalitions were trying to do rapid research to understand “when does public health concern around spreading the disease override some of these federal funding requirements?” Based on these inconsistencies, coalition executive directors felt they could talk about the requirements for isolation and quarantine, as well as the benefits of testing, wearing masks, and later vaccines, but they did not feel confident navigating the “matrix of rules, Federal funding, and public health approaches, while also dealing with staff shortages.”

### Closure of schools, workplaces, and essential and non-essential services

Many executive directors talked about the advantages of moving to virtual platforms for victims when schools, workplaces, and even essential services went virtual, but they also identified downsides. “When court hearings switched to a virtual platform, that was extremely tricky for a survivor who may have been under a stay-at-home order with their abuser.” Even when victims were not sharing the same space as an abuser, some feared that an abuser could “know by my Zoom picture where I’m at or who I’m staying with and come after me.” Once courts did reopen, there were long backlogs “particularly in family law around divorce and custody issues …so people who are trying to get divorced from their abuser can't get into court.” Virtual court also often meant that victims needed to appear without advocates. Even in essential settings that remained open during the pandemic, such as hospitals, in person accompaniment was completely stopped, even for people “that might be domestic violence, sexual assault, or trafficking victims …where normally we would go to the hospital and advocate for them twenty-four seven, that has stopped.”

School and community center closures presented a number of challenges to domestic violence service providers. Internet access, trainings about how to use Zoom, and limited access to updated computers with cameras were all challenges for children in shelters or hotels who needed to attend school virtually. The closure of schools and community centers, including churches and synagogues, also limited domestic violence service providers ability to do community education. In many cases, these programs did not restart reopening these facilities due to the lack of interest in being part of large group meeting or other limitations that remained in place on gatherings.

The closure of workplaces also had severe impacts on the provision of domestic violence advocacy and resources services. Technologies for remote work – for clients and for coalition employees, shelter staff, and other advocates – required not only updated equipment, software, and training, but also raised questions about the “security and confidentiality” of these systems. Cell phones frequently replaced office lines, but service was not always available for victims, programs, or staff, particularly those living in rural areas. Providing training and other services virtually also often required service providers and advocates to purchase new technology – cell phones, web cameras, laptops with cameras.

The closure of non-essential services or the ability of non-essential staff to work remotely caused immediate and deep divisions among some domestic violence service provider staff. “Overnight staff and shelter staff …had to go in …to get their job done,” which led to tensions inside agencies around policies and practices. The lack of hazard pay, or even any policies around who may qualify for hazard pay, made shelter staff feel undervalued, even when being asked to come to work in person in a potentially dangerous situation. As one coalition executive director summarized, “agencies thought, ‘we have to do something to compensate them because it doesn't seem fair that some staff can work at home and others are now in jeopardy’ especially at the beginning of the pandemic.”

### Personal protective equipment

Domestic violence service providers and advocates were deeply impacted by the initial shortages of PPE and other essential supplies like cleaning products, diapers, baby formula, and toilet paper. However, even once masks and other PPE were more widely available, confusion remained about the ability to require them given conflicting public health guidance and federal funding guidelines under VAWA. Further, implementing mandates, particularly if done without adequate education and risk communication, potentially disempowered victims and were not trauma-informed. Donations of essential supplies to shelters ebbed and flowed and oftentimes were redirected due to confusion about domestic violence service providers and advocates’ status as non-essential workers. One coalition executive director summarized this dilemma passionately, pointing out that the domestic violence workforces “really were invisible first responders” during the pandemic. The respondent goes on to say “nobody ever talks about advocates being first responders but we really are because people in crisis come to us sometimes before engaging any of the other formal first responders. But we didn't have masks, we didn't have gloves, we didn't have the barrier up. We had nothing and it took a long time because that [stuff] was in short supply everywhere.”

## Discussion

Our findings largely support the published literature, which demonstrates that domestic violence victims, service providers, and advocates faced a myriad of challenges with the implementation of non-pharmaceutical control measures over the course of the COVID-19 pandemic [7]. Travel restrictions and stay-at home orders issued by elected officials and public health authorities required residents to stay at home except for essential activities, such as obtaining food and medication, or essential work, such as healthcare or infrastructure operations [17]. Isolation, financial strain, and psychological stressors associated with travel restrictions and stay-at-home orders increased the vulnerability of individuals to domestic violence. Once stay-at-home orders were relaxed, ongoing physical distancing requirements frequently required a shift from face-to-face to phone or online/remote service delivery. As face-to-face contact with clients decreased, there were some advantages (e.g., immediate response and support through online chats and phone calls), but there were also disadvantages, including added stress and extra work for practitioners, as well as concerns about the sustainability of remote service delivery and the effectiveness of virtual services for clients with limited access to, and proficiency with, technology [18, 19].

Research has also pointed out the need to train new types of community-based advocates – like food bank and other frontline workers – to recognize the signs of domestic violence and be trained to provide resources to victims [20]. In addition, more advocates from certain communities may be needed, for example to address the increases in physical violence targeting Asian minority groups perceived to be responsible for carrying the virus. Enhanced training and skills are also needed to maintain the most effective and important virtual services, such as those that reduce stigma and other barriers to services, going forward [21]. Development and implementation of necessary training, technology, and evaluation of virtual services would need to be incorporated into disaster preparedness planning. This expanded training could be particularly important in disasters and emergencies, during which access to typical support, facilities, and resources may be limited while the need for these services is greatly increased.

During the first year of the pandemic, any patient testing positive was required to isolate for 10 days, while close contacts were required to quarantine for 14 days. Although services could still be provided remotely to victims in isolation or quarantine, providers were concerned about the safety of virtual services for clients who were isolated with their abuser [18]. In fact, victims did report that abusers used technology to monitor, surveil, and intimidate [22]. The isolation and quarantine guidelines necessary to limit the spread of COVID-19 pandemic overlapped extensively with risk factors for domestic violence [23]. Changing family dynamics, the result of income loss, unemployment, and rising tensions due to closure of workplaces, schools, and non-essential services meant a loss of flexibility for some and a loss of access to a range of nutritional, physical, and mental health services for others [24, 25]. Exacerbated tension [24] and parental stress [26] also likely increased the risk of family violence. Increases in demand for services left providers and advocates struggling to balance their concerns for personal safety with their obligation to provide accommodation, advocacy, and support without proper PPE or paid time off [27]. Many domestic violence programs did not have adequate PPE to provide services safely or the flexibility in funding to acquire cleaning supplies, masks, or gloves for staff or survivors or to provide hazard pay for those required to work on site, such as shelter staff [28]. A disaster response plan that addresses the need for many types of flexible resources, be they hotel rooms, hazard pay, or smartphones, is needed [21].

This study had several important limitations. Only executive directors of U.S. state and territorial coalitions were interviewed and thus the findings reported here do not directly represent the experiences of local shelters and programs. However, a follow-up online survey of agency leaders is currently underway. While each coalition performs a set of essential functions as defined by NNEDV [29], each coalition is also unique. Thus, even a relatively robust sample would not be nationally representative, particularly if there was response bias (e.g., if those who agreed to participate were in coalitions that differed from those that did not participate). We did not identify the states and territories that did respond to protect confidentiality. Due to the decentralization of the public health response to the pandemic, each state adopted different control measures at different time points and relatedly had different rates of COVID-19 cases at different waves of the pandemic. We did not attempt to determine if there were correlations between information reported by respondents and the status of COVID-19 cases or control measures; however, 13 of 25 (52%) of participants were from “blue” states won by President Biden in the 2020 U.S. Presidential election. Finally, the key informant interview guide did not directly address policy implications for future disasters or pandemics.

In response to the unprecedented challenge of the pandemic, domestic violence coalition staff, as well as direct service providers and advocates used creative problem solving to build resilience within systems that were already operating beyond capacity pre-pandemic. Shelters and other programs stayed open, and domestic violence coalitions and advocates made it a priority to provide trauma-informed services where and when survivors need them, even when physical spaces or interactions were limited or closed. However, system- and policy-level changes are needed to codify the ongoing support of this ad hoc problem solving. Factors including “training, licensing, safety, privacy, payment, and evaluation” will be required to sustain the rapid and unplanned expansion of services [21]. Policies that formalize flexible funding, as well as policies related to other social determinants of health, such as eviction moratoriums, will also be necessary [8].

Coalition executive directors universally agreed there were advantages to adaptions made to meet the public health requirements that were part of the response to the COVID-19 pandemic. For example, virtual and remote work and other systems made access to telehealth and telecounseling easier, especially for victims with children, childcare issues, or family members who were not eligible to be vaccinated or part of vulnerable groups. As one respondent put it, “getting creative on how you serve people has been a big boon for us and for the direct service programs …to really figure out how you're going to serve somebody virtually, how you can meet somebody in person and still stay safe.” Complying with public health control measures challenged domestic violence service providers and victims, yet successes that met needs can be identified and carried forward through preparedness planning and policymaking. Coalitions are now tasked with identifying what adaptations were effective and how those adaptations should be continued in order to increase social services systems’ resilience to future public health emergencies and other types of disasters. Further, coalitions must assess how amended continuity of operations measures can be adequately staffed, funded, and evaluated as part of the evidence base for survivor-centered and empowerment-focused services.

## Conclusion

In many cases, disasters or emergencies may require the adoption of public health control measures to protect public health. These measures may have unintended consequences such as limiting the capacity of and access to services or exacerbating social isolation and stigma. As rates of domestic violence increase across the U.S. and globally, it is important for services to remain open and able to adapt to operations during any type of disaster or emergency. Further, services must accommodate the control measures implemented to reduce the emergency’s overall impact. This means ensuring domestic violence service providers and advocates can access proper PPE, be paid commensurately for their increased labor, and have support systems within, and outside of, their agencies that can support their mental and physical well-being [27]. Advocates have found new solutions in response to the challenges of the COVID-19 pandemic; lessons learned must be applied to keep up with expected increased demand for services.

### Supplementary Information

**Additional file 1.**

## Data Availability

Datasets generated and/or analysed during the current study include only transcripts of the interviews. These datasets are not publicly available as confidentiality is essential related to issues pertaining to domestic violence. Deidentified data may be available from the corresponding author on reasonable request.
