# Supplementary material for: Characterizing the impacts of public health control measures on domestic violence services: qualitative interviews with domestic violence coalition leaders
Source: BMC Public Health. 2023 Sep 5;23:1721. doi: 10.1186/s12889-023-16471-4 (PMC10478408; doi:10.1186/s12889-023-16471-4)
Supplement: Supplementary file 1 — Additional file 1. [file 12889_2023_16471_MOESM1_ESM.docx]

**Appendix 1. Semi-structured interview guide**

1. In your opinion, how did COVID-19 and the response to it create barriers in your state to providing sheltering and other advocacy services - like court support, visitation, batterer intervention?
2. Due to measures like social distancing or closures of non-essential services, what areas of system advocacy did you find your coalition engaging **MORE** in (could prompt with hotel vouchers because shelters were full due to social distancing)?

Were there areas where you did **LESS** engagement due to COVID?

1. What do you think were the most critical unmet needs of the shelter programs in your state or territory during the COVID-19 pandemic?
2. What kinds of funding shifts occurred? How did that impact survivors and services?

We want to provide you with a final opportunity to let us know about other barriers that we may not have asked about:

1. Were there other barriers - created by COVID-19 or the response to it - that shelters or service providers faced? (Prompt with issues like: staffing shortages due to children out of school or fear of exposure; financial shortages due to extra expenses associated with need for masks or cleaning products)
2. Are there takeaways from this experience that can be carried forward to regular operations or emergency responses? (Prompt: Use of technology like texting
3. Are there other people at your coalition or within your professional networks that you suggest we talk to about this topic?
